# Supplementary material for: Next Generation Health Claims Based on Resilience: The Example of Whole-Grain Wheat
Source: Nutrients. 2020 Sep 25;12(10):2945. doi: 10.3390/nu12102945 (PMC7599623; doi:10.3390/nu12102945)
Supplement: Supplementary file 1 [file nutrients-12-02945-s001.pdf]

Supplemental table 1 Overview of proposed claims for grain products

Nutrient substance, food or food category	Claim	Conditions of use of the claim / Restrictions of use / Reasons for non-authorisation	Health relationship
**Rye fibre**	Rye fibre contributes to normal bowel function	The claim may be used only for food which is high in that fibre as referred to in the claim HIGH FIBRE as listed in the Annex to Regulation (EC) No 1924/2006.	changes in bowel function
**Rye bread**	-Stimulates insulin secretion.<br><br>-Low insulin response.	Non-compliance with the Regulation because on the basis of the scientific evidence assessed, this food is not sufficiently characterised for a scientific assessment of this claimed effect and the claim could not therefore be substantiated.	not validated
**Rye fibre**	Helps to maintain healthy cholesterol level.<br><br>Brand name which contains the claim: Sydänystävä<br><br>"Friend of the heart".<br><br>Clarification provided<br><br>Helps to maintain healthy cholesterol level. Brand name which contains the claim: Sydänystävä "Friend of the heart".	Non-compliance with the Regulation because on the basis of the scientific evidence assessed, this claimed effect for this food has not been substantiated.	maintenance of normal blood LDL-cholesterol concentrations
**Rye fibre**	Long-lasting energy.<br><br>Levels out the blood sugar increase after meals.<br><br>Low glycemic index.	Non-compliance with the Regulation because on the basis of the scientific evidence assessed, this claimed effect for this food has not been substantiated.	reduction of post-prandial glycaemic responses
**Rye flour**	Rukkijahu iseloomustab madal glükeemiline indeks.<br><br>Clarification provided<br><br>Rye flower is characterised by low glycaemic index.	Non-compliance with the Regulation because on the basis of the scientific evidence assessed, this food is not sufficiently characterised for a scientific assessment of this claimed effect and the claim could not therefore be substantiated.	not validated
**High-fibre sourdough rye bread**	Reduction of post-prandial glycaemic responses compared with glucose	Non-compliance with the Regulation because on the basis of the scientific evidence assessed, this claimed effect for this food has not been substantiated.	
**Wheat bran fibre**	Wheat bran fibre contributes to an acceleration of intestinal transit	The claim may be used only for food which is high in that fibre as referred to in the claim HIGH FIBRE as listed in the Annex to Regulation (EC) No 1924/2006. In order to bear the claim information shall be given to the consumer that the claimed effect is obtained with a daily intake of at least 10 g of wheat bran fibre.	reduction in intestinal transit time
**Wheat bran fibre**	Wheat bran fibre contributes to an increase in faecal bulk	The claim may be used only for food which is high in that fibre as referred to in the claim HIGH FIBRE as listed in the Annex to Regulation (EC) No 1924/2006.	Increase in faecal bulk
**Wheat Dextrin**	- Diet rich in fiber can help you maintain good cardiovascular health.<br><br>- Dietary fiber helps maintain healthy cholesterol	Non-compliance with the Regulation because on the basis of the scientific evidence assessed, this claimed effect for this food has not been substantiated.	maintenance of normal (fasting) blood

	levels to promote overall heart health.<br><br>- Dietary fiber helps maintain healthy blood pressure to promote overall heart health.<br><br>-Diets rich in fiber can help promote healthy triglyceride levels.		concentrations of triglycerides
**Wheat Dextrin**	- Diet rich in fiber can help you maintain good cardiovascular health.<br><br>- Dietary fiber helps maintain healthy cholesterol levels to promote overall heart health.<br><br>- Dietary fiber helps maintain healthy blood pressure to promote overall heart health.<br><br>-Diets rich in fiber can help promote healthy triglyceride levels.	Non-compliance with the Regulation because on the basis of the scientific evidence assessed, this claimed effect for this food has not been substantiated.	maintenance of normal blood cholesterol concentrations
**Wheat Dextrin**	- Diet rich in fiber can help you maintain good cardiovascular health.<br><br>- Dietary fiber helps maintain healthy cholesterol levels to promote overall heart health.<br><br>- Dietary fiber helps maintain healthy blood pressure to promote overall heart health.<br><br>-Diets rich in fiber can help promote healthy triglyceride levels.	Non-compliance with the Regulation because on the basis of the scientific evidence assessed, this claimed effect for this food has not been substantiated.	maintenance of normal blood pressure
**Melon extract (containing SOD) /Wheat Gliadin**	- Protects organism against effects of the free radicals in excess during oxidative stress.<br><br>- Protects against cellular ageing induced by free radicals	Non-compliance with the Regulation because on the basis of the scientific evidence assessed, this claimed effect for this food has not been substantiated.	protection of DNA, proteins and lipids from oxidative damage
**Melon extract (containing SOD) /Wheat Gliadin**	- Quenches excess free radicals such as from pollutants<br><br>- The anti-ageing catalyst<br><br>- Fights premature ageing<br><br>- Fights the signs of premature ageing<br><br>- The anti-premature ageing catalyst	Non-compliance with the Regulation because on the basis of the scientific evidence assessed, this claimed effect for this food has not been substantiated.	protection of DNA, proteins and lipids from oxidative damage

**Melon extract (containing SOD) /Wheat Gliadin**	- Quenches excess free radicals such as from pollutants<br><br>- The anti-ageing catalyst<br><br>- Fights premature ageing<br><br>- Fights the signs of premature ageing<br><br>- The anti-premature ageing catalyst	Non-compliance with the Regulation because on the basis of the scientific evidence assessed, this claimed effect for this food has not been substantiated.	protection of the skin from photo-oxidative (UV-induced) damage
**Melon extract (containing SOD) /Wheat Gliadin**	- Reinforces the body's own natural defences.<br><br>- Promotes the whole natural defences against free radicals in excess<br><br>- Helps maintain the immune system	Non-compliance with the Regulation because on the basis of the scientific evidence assessed, this claimed effect for this food has not been substantiated.	protection of DNA, proteins and lipids from oxidative damage
**Melon extract (containing SOD) /Wheat Gliadin**	- Reinforces the body's own natural defences.<br><br>- Promotes the whole natural defences against free radicals in excess<br><br>- Helps maintain the immune system	Non-compliance with the Regulation because on the basis of the scientific evidence assessed, this claimed effect for this food is not sufficiently defined to be able to be assessed and the claim could not therefore be substantiated.	"effects on immune system"
**Wheat Dextrin**	- Wheat dextrin has low glycemic and insulinemic indices and it is suitable for use by diabetics.<br><br>- Eating a diet high in fiber can help control postprandial glucose levels and serum lipid profiles.<br><br>- Dietary fiber helps improve glycemic control to improve your ability to maintain normal blood sugar and insulin levels, essential for good health	Non-compliance with the Regulation because on the basis of the scientific evidence assessed, this claimed effect for this food has not been substantiated.	reduction of post-prandial glycaemic responses
**Wheat Dextrin**	-Diet rich in fiber can help you maintain good cardiovascular health.<br><br>-Dietary fiber helps maintain healthy cholesterol levels to promote overall heart health.<br><br>-Dietary fiber helps maintain healthy blood pressure to promote overall heart health.<br><br>-Diets rich in fiber can help promote healthy triglyceride levels	Non-compliance with the Regulation because on the basis of the scientific evidence assessed, this claimed effect for this food has not been substantiated.	maintenance of normal (fasting) blood concentrations of triglycerides
**Wheat Dextrin**	-Diet rich in fiber can help you maintain good cardiovascular health.	Non-compliance with the Regulation because on the basis of the scientific evidence assessed, this claimed effect for this food has not been substantiated.	maintenance of normal blood

	-Dietary fiber helps maintain healthy cholesterol levels to promote overall heart health.<br><br>-Dietary fiber helps maintain healthy blood pressure to promote overall heart health.<br><br>-Diets rich in fiber can help promote healthy triglyceride levels		cholesterol concentrations
**Wheat Dextrin**	-Diet rich in fiber can help you maintain good cardiovascular health.<br><br>-Dietary fiber helps maintain healthy cholesterol levels to promote overall heart health.<br><br>-Dietary fiber helps maintain healthy blood pressure to promote overall heart health.<br><br>-Diets rich in fiber can help promote healthy triglyceride levels	Non-compliance with the Regulation because on the basis of the scientific evidence assessed, this claimed effect for this food has not been substantiated.	maintenance of normal blood pressure
**Wheat Dextrin**	-Wheat dextrin has low glycemic and insulinemic indices.<br><br>-Eating a diet high in fiber can help control postprandial glucose levels and serum lipid profiles.<br><br>-Dietary fiber helps improve glycemic control to improve your ability to maintain normal blood sugar and insulin levels, essential for good health.	Non-compliance with the Regulation because on the basis of the scientific evidence assessed, this claimed effect for this food has not been substantiated.	reduction of post-prandial glycaemic responses
**Wheat germ oil**	1. Improves metabolism<br><br>2. Helps maintain functions and structure of the body and its organ cell membranes<br><br>3. Activates absorption of calcium in intestinal tract thereby increasing bone density<br><br>4. Promotes body purification and reduction of body mass	Non-compliance with the Regulation because on the basis of the scientific evidence assessed, this claimed effect for this food has not been substantiated.	maintenance of normal bone
**Wheat germ oil**	1. Improves skin condition, its elasticity and firmness, promotes natural renewal of skin cells, normalizes skin's moisture level by moisturizing it in a natural way.	Non-compliance with the Regulation because on the basis of the scientific evidence assessed, this claimed effect for this food has not been substantiated.	maintenance of normal skin hydration

**Wheat germ oil**	1. Increases immunity<br><br>2. Ensures activity of the immune system, reduction of inflammatory reactions	Non-compliance with the Regulation because on the basis of the scientific evidence assessed, this claimed effect for this food is not a beneficial physiological effect as required by the Regulation.	reduction of inflammation
**Wheat germ oil**	1. Strengthens the cardiovascular system<br><br>2. Promotes lmaintenance of vascular elasticity, heart health and normal blood pressure<br><br>3. Ensures regulation of the blood vessel tonus	Non-compliance with the Regulation because on the basis of the scientific evidence assessed, this claimed effect for this food has not been substantiated.	maintenance of normal blood pressure
**Wheat (Triticum vulgare)**	Contributes to maintain a healthy skin.<br><br>Contributes to skin hydration/ moisturizing.<br><br>Helps to protect the skin.<br><br>Helps to support skin's natural defenses against UV-radiations.<br><br>Antioxidants help your skin to combat the production of free radicals during exposure to the sun.	Non-compliance with the Regulation because on the basis of the scientific evidence assessed, this claimed effect for this food has not been substantiated.	maintenance of normal skin hydration
**Wheat (Triticum vulgare)**	Contributes to maintain a healthy skin.<br><br>Contributes to skin hydration/ moisturizing.<br><br>Helps to protect the skin.<br><br>Helps to support skin's natural defenses against UV-radiations.<br><br>Antioxidants help your skin to combat the production of free radicals during exposure to the sun.	Non-compliance with the Regulation because on the basis of the scientific evidence assessed, this claimed effect for this food has not been substantiated.	protection of the skin from photo-oxidative (UV-induced) damage
**Cystine (wheat extract)**	Contributes to normal hair growth	Non-compliance with the Regulation because on the basis of the scientific evidence assessed, this claimed effect for this food has not been substantiated.	maintenance of normal hair
**Wheat**<br><br>**(Triticum vulgare)**	Helps to control blood levels of cholesterol.<br><br>Contributes to a healthy cholesterol level and healthy blood vessels.<br><br>Contributes to a normal blood pressure.<br><br>Helps to maintain a healthy heart.	Non-compliance with the Regulation because on the basis of the scientific evidence assessed, this claimed effect for this food has not been substantiated.	maintenance of normal blood cholesterol concentrations

	Contributes to normal cholesterol levels.<br><br>Helps to reduce blood cholesterol levels.		
**Wheat**<br><br>**(Triticum vulgare)**	Helps to control blood levels of cholesterol.<br><br>Contributes to a healthy cholesterol level and healthy blood vessels.<br><br>Contributes to a normal blood pressure.<br><br>Helps to maintain a healthy heart.<br><br>Contributes to normal cholesterol levels.<br><br>Helps to reduce blood cholesterol levels.	Non-compliance with the Regulation because on the basis of the scientific evidence assessed, this claimed effect for this food has not been substantiated.	maintenance of normal blood pressure
**Wheat grain fibre**	Helps with weight control	Non-compliance with the Regulation because on the basis of the scientific evidence assessed, this claimed effect for this food has not been substantiated.	contribution to the maintenance or achievement of a normal body weight
**Wheat germ oil**	Increases potency	Non-compliance with the Regulation because on the basis of the scientific evidence assessed, this claimed effect for this food has not been substantiated.	contribution to normal fertility
**Wheat Dextrin**	Increasing fiber intake helps maintain digestive health.<br><br>Wheat dextrin promotes healthy functioning of your digestive system.<br><br>Wheat dextrin helps your natural bowel regularity.<br><br>Wheat dextrin helps to restore normal digestive health.<br><br>Wheat dextrin is a natur	Non-compliance with the Regulation because on the basis of the scientific evidence assessed, this claimed effect for this food has not been substantiated.	maintenance of normal bowel function
**Wheat Dextrin**	Increasing fiber intake helps maintain digestive health.<br><br>Wheat dextrin promotes healthy functioning of your digestive system.<br><br>Wheat dextrin helps your natural bowel regularity.<br><br>Wheat dextrin helps to restore normal digestive health.	Non-compliance with the Regulation because on the basis of the scientific evidence assessed, this claimed effect for this food has not been substantiated.	decreasing potentially pathogenic gastro-intestinal microorganisms

	Wheat dextrin is a natural solution when it concerns your digestive health.<br><br>Wheat dextrin helps to supplement your daily diet with fiber that is essential to keep your bowel healthy.<br><br>Wheat dextrin helps restore your digestive system's natural balance.		
**Wheat Dextrin**	Increasing fiber intake helps maintain digestive health.<br><br>Wheat dextrin promotes healthy functioning of your digestive system.<br><br>Wheat dextrin helps your natural bowel regularity.<br><br>Wheat dextrin helps to restore normal digestive health.<br><br>Wheat dextrin is a natural solution when it concerns your digestive health.<br><br>Wheat dextrin helps to supplement your daily diet with fiber that is essential to keep your bowel healthy.<br><br>Wheat dextrin helps restore your digestive system's natural balance.	Non-compliance with the Regulation because on the basis of the scientific evidence assessed, this claimed effect for this food has not been substantiated.	maintenance of normal bowel function
**Wheat germ oil**	Necessary for normal growth, wholesome mental and physical development, a healthy nervous system, maintenance of memory and cognitive abilities, particularly in old age	Non-compliance with the Regulation because on the basis of the scientific evidence assessed, this claimed effect for this food has not been substantiated.	contribution to normal cognitive function
**Melon extract (containing SOD) /Wheat Gliadin**	Quenches excess free radicals such as from pollutants<br><br>- The anti-ageing catalyst<br><br>- Fights premature ageing<br><br>- Fights the signs of premature ageing<br><br>- The anti-premature ageing catalyst	Non-compliance with the Regulation because on the basis of the scientific evidence assessed, this claimed effect for this food has not been substantiated.	protection of the skin from photo-oxidative (UV-induced) damage
**Wheat germ oil**	Relieves painful menstruation	Non-compliance with the Regulation because on the basis of the scientific evidence assessed, this claimed effect for this food has not been substantiated.	relief of menstrual pain

**Wheat germ oil**	Retards the ageing processes	Non-compliance with the Regulation because on the basis of the scientific evidence assessed, this claimed effect for this food is not sufficiently defined to be able to be assessed and the claim could not therefore be substantiated.	protection of cells from premature aging
**Wheat germ oil**	Strengthens the digestive system.	Non-compliance with the Regulation because on the basis of the scientific evidence assessed, this claimed effect for this food is not sufficiently defined to be able to be assessed and the claim could not therefore be substantiated.	"digestive system"
**Wheat germ oil**	Strengthens the nervous system	Non-compliance with the Regulation because on the basis of the scientific evidence assessed, this claimed effect for this food has not been substantiated.	contribution to normal neurological function
**Wheat Dextrin**	Wheat dextrin helps to improve the absorption of calcium and magnesium; two minerals that are essential for healthy muscles and bones.<br><br>Wheat dextrin improves magnesium and calcium absorption and retention.<br><br>Wheat dextrin helps increase the absorption and retention of certain vital nutrients and promotes overall good health.	Non-compliance with the Regulation because on the basis of the scientific evidence assessed, this claimed effect for this food has not been substantiated.	increase in magnesium and/or calcium retention
**Wheat Dextrin**	Wheat dextrin is fermented in the gut leading to the production of the beneficial SCFA.<br><br>Wheat dextrin helps promote colon health.<br><br>Wheat dextrin nourishes the digestive tract, where 70% of immune function occurs.<br><br>Wheat dextrin stimulates the microflora to restore and maintain digestive system's healthy balance.<br><br>Wheat dextrin stimulates the microflora (prebiotic effect).	Non-compliance with the Regulation because on the basis of the scientific evidence assessed, this claimed effect for this food has not been substantiated.	decreasing potentially pathogenic gastro-intestinal microorganisms
**Wheat Dextrin**	Wheat dextrin is fermented in the gut leading to the production of the beneficial SCFA.<br><br>Wheat dextrin helps promote colon health.<br><br>Wheat dextrin nourishes the digestive tract, where 70% of immune function occurs.<br><br>Wheat dextrin stimulates the microflora to restore and maintain digestive system's healthy balance.	Non-compliance with the Regulation because on the basis of the scientific evidence assessed, this claimed effect for this food is not a beneficial physiological effect as required by the Regulation.	short chain fatty acid (SCFA) production in the bowel

	Wheat dextrin stimulates the microflora (prebiotic effect).		
**Wheat polar lipid extract**	Contributes to improve skin hydration	Non-compliance with the Regulation because on the basis of the scientific evidence assessed,this claimed effect for this food has not been substantiated	
**Arabinoxylan produced from wheat endosperm**	Consumption of arabinoxylan as part of a meal contributes to a reduction of the blood glucose rise after that meal	The claim may be used only for food which contains at least 8 g of arabinoxylan (AX)-rich fibre produced from wheat endosperm (at least 60 % AX by weight) per 100 g of available carbohydrates in a quantified portion as part of the meal. In order to bear the claim information shall be given to the consumer that the beneficial effect is obtained by consuming the arabinoxylan (AX)-rich fibre produced from wheat endosperm as part of the meal.	reduction of post-prandial glycaemic responses
**Wheat sprouts**	For eye health.	Non-compliance with the Regulation because on the basis of the scientific evidence assessed, this claimed effect for this food has not been substantiated.	maintenance of normal vision
**Wheat sprouts**	Strong plant antioxidant. Protect cells from premature ageing.	Non-compliance with the Regulation because on the basis of the scientific evidence assessed, this claimed effect for this food is not a beneficial physiological effect as required by the Regulation.	Antioxidant, antioxidant content, and antioxidant properties
**Wheat sprouts**	Strong plant antioxidant. Protect cells from premature ageing.	Non-compliance with the Regulation because on the basis of the scientific evidence assessed, this claimed effect for this food is not sufficiently defined to be able to be assessed and the claim could not therefore be substantiated.	Protection of cells from premature aging
**Whole grain foods**	People who eat more whole grain foods tend to have a healthier body weight and gain less weight over time (as part of a low fat diet & healthy lifestyle).	Non-compliance with the Regulation because on the basis of the scientific evidence assessed, this food is not sufficiently characterised for a scientific assessment of this claimed effect and the claim could not therefore be substantiated.	not validated
**Whole grain, whole grain flour**	Täisteratoodete tarbimine reguleerib vere kolesteroolitaset.<br><br>Clarifications provided<br><br>Consuming whole grain products regulates blood cholesterol level.	Non-compliance with the Regulation because on the basis of the scientific evidence assessed, this food is not sufficiently characterised for a scientific assessment of this claimed effect and the claim could not therefore be substantiated.	not validated
**Whole grain, whole grain flour**	Täisteratoodete tarbimine soodustab seedimist. Consumption of whole grains helps promote digestion.	Non-compliance with the Regulation because on the basis of the scientific evidence assessed, this food is not sufficiently characterised for a scientific assessment of this claimed effect and the claim could not therefore be substantiated.	not validated
**Whole grain, whole grain flour**	Täisteratoodete tarbimine suurendab küllastustunnet ehk täiskõhutunnet.<br>Täisteratoodete tarbimine pikendab küllastustunde ehk täiskõhutunde säilimist.<br>Clarifications provided<br>Consuming whole grain products increases satiety.<br>Consuming whole grain products prolongs the feeling of satiety.	Non-compliance with the Regulation because on the basis of the scientific evidence assessed, this food is not sufficiently characterised for a scientific assessment of this claimed effect and the claim could not therefore be substantiated.	not validated
**Whole grain, whole grain flour**	Täisteratooteid iseloomustab madal glükeemiline indeks.<br>Clarifications provided	Non-compliance with the Regulation because on the basis of the scientific evidence assessed, this food is not sufficiently characterised for a scientific assessment of this claimed effect and the claim could not therefore be substantiated.	not validated

	Whole grain products are characterised by low glycaemic index		
**Diet rich in whole grain**	diets rich in whole grain foods promote heart health	Non-compliance with the Regulation because on the basis of the scientific evidence assessed, this food is not sufficiently characterised for a scientific assessment of this claimed effect and the claim could not therefore be substantiated.	not validated
**Carbohydrate sources with low glycaemic index (GI <55), e.g. legumes, vegetables, whole grain cereals**	Consumption of low GI (low glycaemic index) foods with in the healthy diet (low glycaemic load - GL) can contribute to the maintenance of the normal blood sugar level, blood lipid level (triglyceride) and body mass.	Non-compliance with the Regulation because on the basis of the scientific evidence assessed, this food is not sufficiently characterised for a scientific assessment of this claimed effect and the claim could not therefore be substantiated.	not validated
**Wholegrain**	Promotes gut activity	Non-compliance with the Regulation because on the basis of the scientific evidence assessed, this food is not sufficiently characterised for a scientific assessment of this claimed effect and the claim could not therefore be substantiated.	not validated
**Wholegrain**	Helps with weight control.<br><br>For a long-lasting sense of satiety.<br><br>Releases energy slowly	Non-compliance with the Regulation because on the basis of the scientific evidence assessed, this food is not sufficiently characterised for a scientific assessment of this claimed effect and the claim could not therefore be substantiated.	not validated
